# Restore politics in societal debates on new genomic techniques

**DOI:** 10.1007/s10460-022-10328-z

**Published:** 2022-07-07

**Authors:** Lonneke M. Poort, Jac. A. A. Swart, Ruth Mampuys, Arend J. Waarlo, Paul C. Struik, Lucien Hanssen

**Affiliations:** 1 Erasmus School of Law, Sociology, Theory and Methodology, Erasmus University Rotterdam, Postbus 1738, 3000 DR Rotterdam, The Netherlands; 2 Energy and Sustainability Research Institute Groningen, University of Groningen, Groningen, The Netherlands; 3 The Netherlands Scientific Council for Government Policy, The Hague, Postbus, 20004, 2500 EA The Netherlands; 4 Freudenthal Institute, Utrecht University, PO Box 85.170, 3508 AD Utrecht, The Netherlands; 5 Centre for Crop Systems Analysis, Plant Sciences Group, Wageningen University and Research, Bornsesteeg 48, Wageningen, Gelderland 6708 PE The Netherlands; 6 Deining Societal Communication & Technology Governance, Peter Scheersstraat 26, 6525 DE Nijmegen, The Netherlands

**Keywords:** New genomic techniques, Doubly unstructured problems, Stakeholder participation, Repoliticization, Participation ethics

## Abstract

End of April 2021, the European Commission published its study on New Genomic Techniques (NGTs). The study involved a consultation of Member States and stakeholders. This study reveals a split on whether current legislation should be maintained or adapted to take account of scientific progress and the risk level of NGT products. This split was predictable. New technological developments challenge both ethical viewpoints and regulatory institutions; and contribute to the growing divide between science and society that value ‘technological innovations’ differently. Such controversies are often characterized as ‘unstructured’ because of nearly unbridgeable positions on entangled scientific and value-laden issues. Initiatives for stakeholder involvement, such as consultation or participation, often focus on reaching a ‘shared vision’ without exploring the diverse societal concerns and values behind these positions. To resolve the EU stalemate in NGT regulation, we advocate to bring back politics in the EU decision-making process instead of hiding it under the veil of science, the need for regulatory change and public support. A more productive and justified use of genuine stakeholder participation is possible, if participants and deliberation design meet the criteria of what we call *participation ethics*. Drawing from our applied experience exploring the ethics of genetic modification, we believe that this approach can lead to more robust political decision-making and restore societal confidence in the governance of contested issues such as NGTs.

## Introduction

New scientific and technological developments, such as New Genomic Techniques (NGTs), do not only challenge our personal and shared ethical viewpoints as well as our legislative and regulatory bodies. They also contribute to the growing divide between science and society that perceive and value ‘technological innovations’ in different ways (Jasanoff 2005). In this context, in April 2021 the European Commission (EC) published the results of its study on New Genomic Techniques. According to the EC, NGTs are techniques capable to alter the genetic material of an organism that have emerged or have been developed over the past two decades, including gene editing. This EC study was requested by the Council of the European Union (EU) to clarify the legal situation of NGTs after a ruling of the European Court of Justice (CJEU) on a technique called mutagenesis.^1^ The EC study comprised two phases of consultation: a consultation of the EU Member States through a specific survey and a targeted stakeholder consultation on the use of NGTs in the different Member States.^2^ A wide variety of stakeholders had been invited, ranging from biotech companies and associations to the organic farming sector and Non-Governmental Organizations (NGOs) (European Commission 2021).

The EC study acknowledges that the current legislation does not keep pace with new biotechnological developments, and, therefore, creates enforcement challenges and legal uncertainties. “*However, reported views are split on whether the current legislation should be maintained, and its implementation reinforced, or rather adapted to take account of scientific and technological progress, the level of risk of NGT products and the benefits to society*” (European Commission 2021 p. 4). The report also identifies knowledge gaps and makes recommendations to address them; moreover, more effort should be made to inform and engage with publics and assess their stances. The Commission ended its report, a bit disappointingly, by calling for further research: “*The follow-up to this study should confirm whether adaptation is needed and, if so, what form it should take and which policy instruments should be used in order for the legislation to be resilient, future-proof and uniformly applied as well as contribute to a sustainable agri-food system*” (European Commission 2021, p. 59).

We consider the call for further research disappointing as the current study did not bring the long-time anticipated progress in the underlying problem of enforcement challenges, legal uncertainties, and social disagreement. These kinds of EU consultation rounds are a recurring phenomenon in the development of biotechnological techniques, whether they be about the genetically modified (GM) micro-organisms (1970s), GM crops and animals (1990s), gene therapy (early 2000s) or, more recently, CRISPR-Cas (2010s) and the possibility of human germline editing (2020s). Nobel Memorial Prize winner and one of the developers of the CRISPR-Cas technique Jennifer Doudna argued that stakeholders must engage in thoughtfully crafting regulations of the technology without stifling it (Doudna 2019). Although we foresee a role for stakeholders, we argue that stakeholder involvement^3^ will not always end the conflict about controversial technologies, neither will it automatically lead to a shared set of rules. More often, it is used strategically as window-dressing avoiding and postponing political decisions on genomic technologies (Poort & Bovenkerk 2016). Policymakers and decision-makers tend to involve stakeholders as a goal, not always as a means to reach towards a certain goal. For stakeholders it, consequently, remains unclear what to expect and how their contributions are used in shaping policy, often resulting in disappointment or feelings of being ignored.

Therefore, we think that initiators of stakeholder involvement should consider and be clear about the goals, as well as the expectations of these activities beforehand.^4^

EU stakeholder consultations strongly focus on identifying the main issues and the dominant positions. For example, the questionnaire used in the EC-study on NGTs merely focused on experiences with the use of NGTs. This strong focus on experiences with the use of NGTS, risks that these underlying viewpoints, worries, unexpected consequences and other sorts of controversies remain hidden. After decades of repetitive debates, it should be clear that ‘facts’ will not resolve the disagreement on NGTs, nor will a consensus emerge from this kind of pre-framed stakeholder activities (Mampuys 2021; De Krom et al. 2014; Hanssen 2009).The rise of controversies around New Plant Breeding Techniques (NPBTs) and now NGTs demonstrates again that new scientific and technological developments do not only challenge our ethical standards and regulatory institutions, but may also contribute to the growing divide between science and society. Lack of public trust in science and emerging technology, sometimes even the denial of scientific insights, is raging in recent years through societies as other controversies illustrate, like the recent COVID-19 vaccine debate (Loomba et al. 2021). Even in cases, where there is agreement among most scientists, such as global warming, both scientific and societal disputes remain (Cook et al., 2016). NGTs may run the risk of becoming just new branches on the tree of societal distrust in science and technology.

In our view, an additional step is needed to move towards political decision-making instead of postponing it. In this article we argue that to guide and facilitate political decision-making, it is essential to make (normative) underlying considerations explicit. We distinguish between two tracks of stakeholder involvement: one with a deliberative dimension and the other with a political dimension. In the deliberative dimension, decisions are not yet made. This dimension primarily focuses on the clarification and articulation of underlying considerations and scientific controversies (Poort & Bovenkerk 2016, p. 282–283). Decisions are made in the political dimension, in which these normative considerations can be incorporated. A critical perspective on stakeholder involvement is not new (see, e.g., Felt & Fochler 2008; Nowotny 2003; Gaskell & Allum 2001; Wynne 1996), neither is differentiation between different tracks or stages of stakeholder involvement (see, e.g., Castle & Culver 2013; Poort 2013; Bovenkerk 2011). However, to make stakeholders involvement in these tracks working, we also need to reconsider the context of stakeholder involvement. We, therefore, formulate an understanding of participation ethics being a set of criteria that need to be met by all participants. In that way, we can avoid risking window-dressing.

## European union discussions on new plant breeding techniques in retrospect

Historically, stakeholder involvement in discussions on NGTs can fit into the picture of a continuous call for expert and stakeholder consultation of what were originally called New Plant Breeding Techniques (NPBTs), to which now also gene editing (CRISPR-Cas) belongs. These new techniques are not only more specific and precise compared with older techniques, but the resulting products (e.g., plants) also cannot be easily distinguished from products created by conventional breeding techniques or from natural variants. Over the years, numerous scientific and legal working groups have been asked to investigate the status of NPBTs and stakeholders have been consulted on their views and asked to participate in consensus-seeking activities. Although framed as a scientific and legal issue, the regulation of these techniques also has a practical (e.g., co-existence with organic farming), normative and ideological side (e.g., the societal organization of the agricultural system). This can partially explain why scientific and legal working groups and commissions issuing reports on NGTs have not resulted in decision-making on their legal status.

For example, the first EU working group on NPBTs was active between 2007 and 2011 and could not reach a consensus on the status of all techniques belonging to it.^5^ Next, the EFSA Genetically Modified Organisms (GMOs) panel adopted scientific opinions on the safety of three techniques: cisgenesis, intragenesis, and Zinc Finger Nuclease (EFSA 2012a, 2012b). Over the years, more techniques were added to the list. Separate expert working groups on synthetic biology were set up upon request from the EC, focusing on the definition, risk assessment methodologies and safety aspects (SCENIHR et al. 2014, 2015a, 2015b). However, none of these processes and reports resulted in decision-making by the EC about the legal status of NPBTs: is it a GMO or not. Instead, more scientific expertise was brought to the table by consulting a newly established advisory body of chief scientific advisors in the ‘Scientific Advice Mechanism’ (SAM). They issued a scoping paper and explanatory note on NPBTs in 2016 and 2017 and a statement on the regulation of gene editing in 2018 (SAM 2016, 2017, 2018).

Mampuys (2021, p. 36) recently illustrated how extensive multi-year stakeholder consultations on GMO safety failed to result in a broadly accepted outcome of the results. She argues that if the underlying debate about normative issues, e.g., safety perceptions, naturalness, and food production processes, is ignored and hidden under the veil of ‘objective science’, all that remains is a never-ending discussion about scientific uncertainty. Earlier, Helliwell et al. (2019) argued that plant genome editing has the potential to become another chapter in the intractable debate that has dogged agricultural biotechnology. Engaged NGOs, like GM Watch, Friends of the Earth or IFOAM Organics Europe, seek to challenge the existing order and broaden the scope of the debate to include also political questions regarding agricultural and technological choices. They bring in other social and ethical dimensions in the agricultural debate in general and in the NGTs discussion. Such NGOs provide alternative and valid ethical and social insights that can open political debate and discussion on the role of emerging technologies, as for example genome editing and the future of agriculture and food sovereignty. Such a repoliticization should be welcomed and engaged with by EU institutions if they are really committed to a wider societal dialogue and more stakeholder and public involvement, as mentioned in the follow-up study by the European Commission.

The EC-study on NGTs seems to be more of the same. As stated in the introduction, stakeholder consultation had a central role in this EC study. At first sight, the distinction between two consecutive phases in this study seemed promising. Both member states and stakeholders, varying from the biotech-industry to the organic sector and NGOs, were requested to share their thoughts, ideas, and experiences. However, as we take a closer look at the questionnaire central in both parts of the study, it merely focused on taking stock of those who work or are willing to work with NGTs. In the questionnaire only two questions were related to ethical concerns, whereas the other 25 questions all focus on gathering information about the use and experiences with NGTs. Furthermore, the first question of the questionnaire already exemplifies the narrow focus by asking the following: “Are your members developing, using, or planning to use NGTs/NGT-products?”^6^ This narrow fact-finding focus did not leave much room for diversity in terms of moral concerns, worries, and other perspectives. Given the history of EU discussion in recent decades we did not expect that the intended Member States and stakeholder consultations will lead to an unambiguous and clearly directed outcome on the preferred legal status of NGTs in Europe.

## The current European Commission consultation round on new genomic techniques

The EC consultation round on NGTs can be seen as yet another case where stakeholder involvement is seemingly overrated, dealing with complex and controversial technological issues, focusing on identifying a ‘shared vision’ without a robust exploration of the diverse societal concerns and conflicting values at stake in a political context. In the study by Mampuys (2021) it is argued that technocratic, regulatory, and even participatory strategies in the context of the GM crop debate are insufficient if political actors, deliberately or not, do not take up their own role. Politicians have the responsibility to *guide* political processes and *take* substantiated and transparent decisions—especially in situations of combined scientific uncertainty and societal disagreement. Currently, we are facing a political void in EU decision-making on GM crops because the political step of decision-making consistently ends in a deadlock in the so-called ‘comitology’ process where no qualified majority in favor or against a draft decision can be reached.^7^ Similarly, decisions on regulatory changes are postponed by means of shifting to other arenas and activities for final answers, such as member state and stakeholder consultations.

The latest study on NGTs by the EC demonstrates again that no EC proposal builds substantially on its results that will easily solve the political deadlock or provide an answer that will satisfy all parties involved. Again, the Commission hangs on filling in knowledge gaps, and starting another round of informing and engaging stakeholders and publics yet postponing the required political step.

“*The GMO legislation has clear implementation challenges and requires contentious legal interpretation to address new techniques and applications. There are strong indications that it is not fit for purpose for some NGTs and their products, and that it needs to be adapted to scientific and technological progress. The follow-up to this study should confirm whether adaptation is needed and, if so, what form it should take and which policy instruments should be used in order for the legislation to be resilient, future-proof and uniformly applied as well as contribute to a sustainable agri-food system*.” (European Commission 2021, p. 60–61).

To resolve the enduring EU stalemate in NGT regulation, we cannot solely build on a technocratic or regulatory strategy alone. Genuine and institutionalized stakeholder participation, instead of only fact-finding consultations, is, therefore, needed. De Krom et al. (2014) already concluded that the institutional treatment of GM field trials is often presented as a single reality with only ‘some epistemological struggles’, turning a multifaceted debate with multiple narratives into a dichotomous one, with proponents and opponents. Also, other authors argued for deliberation that includes other (lay) experts, besides techno-scientists, for answering the question ‘what we can learn about how socio-technological innovations can contribute to sustainable development’. Including those perspectives will broaden deliberation and will avoid dichotomous key political questions concerning controversial techno-scientific experiments (Mampuys 2021; Burall 2018; Inghelbrecht et al. 2017; Hanssen & Gremmen 2013).

Currently, we still witness a technocratic regulatory gaze in the organization of EU stakeholder activities that works in favor of its proponents. Moreover, the framing of the proponents tends to dismiss opponents as 'unscientific' and therefore not legitimate, even when the validity of their own arguments has not been proven yet. Too often it is said that agricultural biotechnology is a necessity for food security, especially in the light of current climate change. Despite the commonly shared, huge optimism about the potential of using transgenics or gene editing techniques, the real evidence for rapid development of resistance to abiotic plant stress is not yet presented (see, e.g., Anwar & Kim 2020). Earlier, Hilbeck et al. (2015) already illustrated that the totality of scientific research outcomes in the field of GM crop safety is nuanced, complex, often contradictory, or inconclusive. Whether to continue and expand the introduction of GM crops are decisions that involve socioeconomic considerations beyond the scope of a narrow scientific debate.

Béné et al. (2019) reviewed the different narratives proposed in literature about sustainable food systems and concluded that the different communities of practice that have engaged in the food system debate diverge in their understanding of the actual nature of the problem and about what the potential solutions are and which one(s) should be given priority. So, this divergence can easily lead to opposition because stakeholders with objections or alternative views on crop breeding and agricultural systems feel being ignored. The problem with agricultural biotechnology therefore is therefore rather sociopolitical than biological in nature (Mueller & Flachs 2021). Thus, besides being careful not to indulge unfounded scientific claims (Stirling 2010; Pielke 2007), participants contributions should also be based on valid ethical and societal arguments (Antonsen & Dassler 2021; Binimelis & Myhr 2016). The initiator of stakeholder involvement should, therefore, carefully consider whom to invite and include based on what we have labelled as ‘participation ethics’, explained below.

Before and after publication of the EC study in April 2021, objections have been put forward in reactions by e.g., *GM Watch*, *Friends of the Earth*, and *IFOAM Organics Europe*.^8^ The last two organizations have participated in the stakeholder survey. Considering the fact-focused nature of the latest EC questionnaire, we are not expecting the second consultation round to bring about a more comprehensive appreciation of NGTs. Underlying viewpoints, worries, unexpected consequences and other sorts of controversies remain hidden. To overcome these dualistic stances, it is important to identify and acknowledge concerns and hopes instead of simply exchanging dualistic standpoints (Poort 2016). In short: there is a strong social, ethical, and economic need for *repoliticization* and for a political readiness to decide on the NGT issues (Purnhagen & Wesseler 2021; Helliwell et al. 2019). Therefore, we argue that making (normative) underlying considerations explicit is essential to guide and facilitate political decision-making. These decisions should not only reflect technological innovation but also a broadly shared vision on 'goods' and 'a good society'.

## Doubly unstructured problems

Inghelbrecht et al. (2017) argued that an evolving technology co-determines the values and structures that form the societal system using it. Technologies-in-use actively shape our interpretation, our action, moral standards, and routines. When trying to understand why a particular technology is favored or strongly opposed, it is therefore important to account for the ethical and social concerns emerging with the use of NGTs in agriculture. Discussions on the use of NGTs in agriculture are of a political nature, touching upon the question how we want to organize EU’s food production. Hence, there is an urgent need to broaden the current mere technocratic discussions with the fundamentally political question of how technology applications co-shape the values and structures that form the agricultural system of the EU. The results should not only reflect technological and regulatory innovation but also a political vision on 'goods' and 'a good society'. This vision should start bottom-up based on the hopes, concerns, and ideals in society.

Controversies on NGTs share a societal confusion, or even discontent, with the perhaps apparently inevitable direction in which society develops through these new technologies without stakeholders and publics having had much say in it. In this context, Jasanoff & Hurlbut (2018) already pointed out that opinions on gene editing in society vary strongly, not only across different societies and communities, but even within them, with the consequence of different understandings of how new technologies should be embedded in society and in legislation. These different understandings should be taken up by politicians, but also scientists and technologists cannot ignore the social and ethical controversies on new scientific insights and novel techniques. Techno-scientific developments often proceed faster than societal and legal debates can cope with. The case of NGTs illustrates this in a dramatic way. This so-called ‘pacing problem’ stresses the need of a framework that includes *public concern assessment* within the professional and conventional ethical and risk assessments to acquire a stronger public and political license for science and technological innovations (Hanssen et al. 2018; Marchant et al. 2011). Recently Macnaghten & Habets (2020) set out a framework of responsible innovation as a tool and an approach to transform the cultures and practices of research itself. They also want a shift from a narrow technical discussion of risks and off-target effects to a wider societal conversation on the stakes underpinning a move into gene-edited crops and foods. A framework of responsible innovation must also be responsive to the wider national and EU political context shaping science policy initiatives. The use of such a framework within the EU political context requires courage and leadership as such a regime will not be in the short-term interests of actors at both sides of the debate.

In this context, a framework suggested by Hisschemöller & Hoppe (1996) may help to arrange the ongoing debates on socio-technocratic controversies, as well as to structure the demand for good governance resulting from it. These authors argue that a problem is *structured* when there is both certainty with respect to scientific knowledge and consensus on relevant values. However, a problem is *unstructured* if both are lacking. If only one of these conditions applies a controversy is considered as *moderately structured*. How should we consider NGTs from this perspective? There is unmistakably much debate on value-related issues of NGTs, such as the rights to produce without GMOs, protection of the environment and the biodiversity, consumer choice, power relations in different markets, and socioeconomic consequences for traditional and organic farming. Besides, there is debate about the scientific status and scientific contributions of NGTs as became clear in the CJEU ruling on mutagenesis. Parties were diametrically opposed on whether new mutagenesis techniques were GM techniques at all (Bergmans et al. 2016). We may therefore consider the case of NGTs as an unstructured problem.

**Fig. 1:**
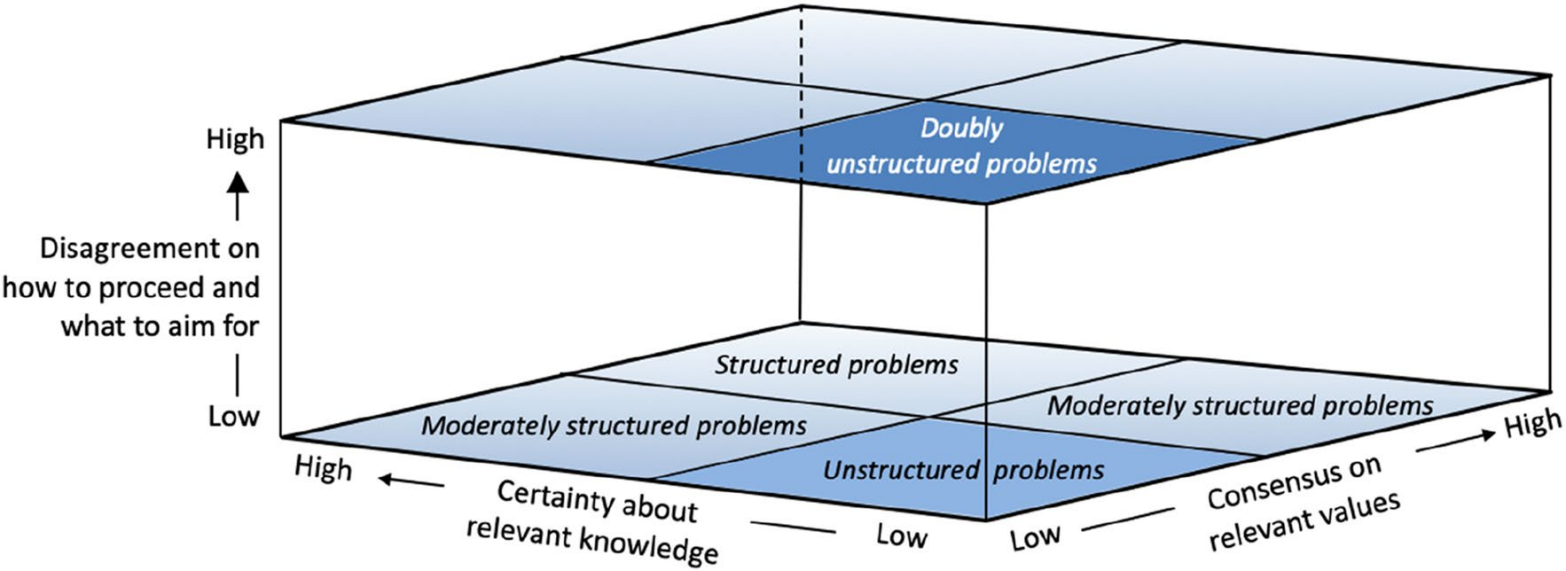
Doubly unstructured problems of science and technology. An unstructured problem arises because of strong disagreement both on relevant knowledge and values that are at stake. A doubly unstructured problem emerges when there is strong disagreement on how to proceed—the governance conditions; and what to aim for—a political vision on ‘goods’ and a ‘good society’—while dealing with an unstructured problem (Hisschemöller & Hoppe 1996)

But it is more complicated than that. Addressing an issue or a controversy as unstructured may be considered as an unstructured problem as it is associated with, often not articulated, perspectives on the nature of scientific claims, social concerns, and political-economic interests at stake, but also the burden of responsibilities carried by science, industry, and politics. It is doubly unstructured. This makes the governance of such a pacing problem, i.e., how to proceed and what to aim for in the controversy, also a matter of dissent and uncertainty. See Fig. 1. The European stalemate situation with respect to New Genomic Techniques may be considered as such a *doubly unstructured problem*.

Our response towards doubly unstructured problems is twofold. First, we do not consider it possible nor useful to strive for a shared substantive vision when dealing with complex issues characterized by uncertainty, rapid technological developments, and a strong moral impact (Poort 2013). Second, we do not think traditional EU consultation activities will do the trick. We do not intend to develop another method for stakeholder involvement that will do so. Much work on methods for participation or consultation has already been done (see. e.g., Castle & Culver 2013; Hagendijk & Irwin 2006; Rowe & Frewer 2005). We do not see a solution in developing another method. We claim, that, instead, stakeholder involvement should start with structuring a problem before defining it. We, therefore, distinguish two tracks for stakeholder involvement relating to both layers in Fig. 1 (Poort 2013, Ch. 10; Bovenkerk & Poort 2008). The first track relates to a first stage of problem-structuring. This stage involves a process of exploring the different layers of the problem at stake, being either structured or unstructured (Hisschemöller and Hoppe, 1996). This track of problem-structuring is followed by a second one, focusing on problem-definition, and on identification﻿ and evaluation of possible policy directions.^9^ The aim of the first track, which we refer to as *the deliberative dimension of stakeholder participation,* would not be for participants to find a shared perspective, but rather to explore the range of different and conflicting value-related societal concerns that may arise from new technological developments. The main goals here are both exploration and articulation of dissents (Castle & Curver 2013; Poort 2013; Harvey 2009)*.*

The second track entails the realm of political decision-making in which participation contributes to demarcation and definition of the policy problem and possible solutions: *the political dimension of stakeholder participation* (Taylor & Dewsbury 2019; Poort 2013; Stirling 2012). Participants of the latter track proceed with the outcomes of the first track, by acknowledging the conclusions, including (minority) diverging viewpoints and underlying arguments. The involved participants may not necessarily be the same ones as in the first track, as deliberation may require a broader range of stakeholders. Therefore, acknowledgment of the outcomes is a relevant step as it contributes to problem-structuring. The output of the political debate should not be confused with a shared consensus on the best way to proceed, but rather focus on a process of balancing and weighing arguments in a transparent and explicit way. This balancing constitutes the basis for the political decision-making process, promoting more substantiation and transparency.

## Participation ethics

The aim of the deliberative dimension of stakeholder participation is not to find a shared perspective, but rather to explore and articulate the range of different and conflicting value-related concerns in society that may arise from new technological developments. It should address questions such as: How may NGTs affect our society and agricultural food production system? Who could be concerned, who are being affected and what kinds of concerns and stakes are likely involved? And, not in the least: what are the underlying values and worldviews that play a role? The latter question has hardly been addressed in the EU consultations. This deliberative dimension requires a public domain, and therefore requires a publicly organized, transparent, open-minded dialogue between scientists, technologists, and representatives of a broad spectrum of civil society organizations. A bottom-up approach may contribute to reveal the complexity of the controversy.

This bottom-up approach, however, can only function adequately, if participants in both tracks are willing to acknowledge and accept controversy, and have a good understanding of their role and the nature of the outcome of the process. We, therefore, introduce ‘participation ethics’, being a set of criteria that need to be met by participants and deliberation design to make a more productive and justified use of genuine stakeholder participation possible. Participation ethics strongly relies on a reciprocal willingness to understand the different views on the matter and an open mind to honestly consider the pronounced pros and cons. The use of vague terms and concepts by participants that cannot be interpreted unambiguously should be avoided (Nuffield 2015). Starting point for such interactions should be a transaction rather than the usual ECs transmission perspective. In ‘communication as transaction’, mutually attribution of meaning and negotiation between participants serve as a basis for interactions. By contrast, ‘communication as transmission’ is based on educating passive publics by experts and so does not account sufficiently for public concerns and questions (Hanssen 2009).

One of the basic grounds of participation ethics concerns reciprocal willingness to participate. ‘Reciprocal willingness’ stems from, amongst others, political and judicial traditions and is used in those contexts to define the relation between an administration and citizens. Where the politicians and/or policymakers may expect citizens to follow the rules, citizens may expect that the administration will abide and apply these rules conscientiously and fairly (Brunnee & Toope 2010). In our opinion, this account of reciprocal willingness is helpful in defining participatory relationships, especially when participants have conflicting value-related concerns. Reciprocal willingness enables that even diametrical opponents may acknowledge they have something to gain from an open deliberation. To that extent, it involves a right to present claims and at the same time have a duty to give reasons for these claims when other participants challenge them (Rawls 1971).

Moreover, in a best-case scenario, reciprocal willingness can result in a process of mutual learning and finding some common ground, where participants not only acknowledge but also appreciate each other's points of view. Different views on definition of the problem, interpretation of research findings and experience-based knowledge, and direction of possible solutions are articulated. The learning that occurs is not so much about 'new facts', but a growing insight in the complexity of the questions: the whole range of points of view and opinions and of opportunities for deliberation and negotiation are taken up (Bull et al. 2008; Chilvers 2008). In our view, participation can be more valuable if it builds towards and on interaction instead of a focus on end results or consensus on ‘the right perspective’ on problem and solution. Building on this account of reciprocity, mutual understanding, and shared ethical standards, we have defined the following criteria for participation ethics.

1. Willingness to clarify and articulate values, worldviews, and interests and to discuss them in a sincere way (no hidden agendas);
2. Preparedness to deal in a respectful way with other positions and to refrain from an authoritative attitude;
3. Willingness to change one’s position and therefore being no longer addressed on a former position after one has publicly changed one’s mind.
  In addition, given one’s commitment to the first three conditions, participants^10^ have:
4. The right to participate or to be represented;
5. And the right to add caveats to the majority viewpoints in the final conclusions of the deliberations.

## Repoliticization in European Union’s decision-making

Based on these considerations, we argue that the mainstream approach of most EU consultation events is insufficient to provide relevant information for this needed process of repoliticization, because it mostly seeks to provide a convergent outcome instead of valuing the richness of different and conflicting perspectives. As an alternative, we have distinguished two tracks for stakeholder participation: one with a deliberative dimension and one with a political dimension. The aim of the deliberative track is not to find a shared perspective, but rather to explore and articulate the range of different and conflicting value-related concerns in society that may arise from new technological developments. The political track focuses on problem-definition by building on problem-structuring as has been done in the first track.

A more productive and justified use of genuine stakeholder participation is possible if participants and deliberation design meet the criteria of what we have called *participation ethics*. In our opinion, the first track or deliberative dimension is an important, but often neglected step in many stakeholder consultations and debates on socio-technological controversies by prematurely focusing on problem-definition and a direct solution to—instead of an exploration of the plethora of (conflicting) ethical and social aspects of—the problem. In the EC-study on NGTs this step was neglected too. The survey held among Member States and among targeted stakeholders was very much focused on the use of NGTs and experiences with the legal framework,^11^ leaving aside broader questions such as the future of agriculture and food security. Taking a step back and opening up a broader debate by first identifying different subjects and stakeholders (See COGEM 2018) may contribute to problem-structuring. The result of deliberation, i.e. a deeper and mutual understanding of the underlying diverging and converging viewpoints and ethical standards, provides input for the second track, the political debate and process. The latter focuses more on defining and deciding the governance conditions of new technologies. EC officials and Member States policymakers involved in EU NGTs politics may attend with an open attitude to all interests and opinions expressed in the deliberative track. In doing so, they can provide support in (re)formulating stakeholder findings and priorities within the existing policy frameworks and political procedures of the EU.

The legitimization of both tracks should be guaranteed in advance to any stakeholder involvement by politicians and/or authorities with a democratically established mandate. The results of the political track should directly inform EU policymakers and politicians involved in decision-making on NGTs. We believe that the approach we propose can lead to more robust political decision-making, because it explicitly presents and addresses the diverging viewpoints that need to be appreciated and balanced together with the scientific state of the art and regulatory context. At the same time, this approach can restore societal confidence in the governance of contested issues such as these NGTs. It is the role and responsibility of politics to decide in situations of (scientific) uncertainty and societal disagreement, instead of opening another round of consultations or falling back on knowledge gaps and legal uncertainties. What the lingering EU debate on NGTs really needs are brave but above all informed politicians who develop a decisive vision on EU’s agricultural system, including the explicit role of NGT’s in this system, and the way to get there. Our proposed *participation ethics* may contribute to a repoliticization in EU’s agriculture decision-making and the possible embedment of new genomic techniques in society.

## Disclaimer

This article is based on discussions in a working group of the subcommittee on Ethics and Societal Aspects of the Netherlands Commission on Genetic Modification (COGEM), in which all authors participated (URL: https://cogem.net/en/). However, the views presented here are those of the authors only and do not necessarily reflect the opinion of the COGEM.

## Abbreviations

CJEU: Court of Justice of the European Union
COGEM: Netherlands Commission on Genetic Modification
EFSA: European Food Safety Authority
SAM: Scientific Advice Mechanism
ScEMA: Subcommittee on Ethics and Societal Aspects
SCENHIR: Scientific Committee on Emerging and Newly Identified Health Risks
SCCS: Scientific Committee on Consumer Safety
SCHER: Scientific Committee on Health and Environmental Risks

## Footnotes

1 Case No 111/18 ruled that organisms obtained by mutagenesis are genetically modified organisms (GMOs) and are, in principle, subject to the obligations laid down by the GMO Directive. For a legal analysis of this case, see Bergmans et al. 2020; Vives-Vallés & Collonnier, 2020.

2 See for an overview: https://ec.europa.eu/food/plant/gmo/modern_biotech/new-genomic-techniques_en

3 In this paper, we use different vocabulary related to stakeholder involvement, such as consultation or participation. We do, however, differentiate between different forms of involvement. We use involvement as an umbrella term. We consider consultation as a means to seek for advice from stakeholders. Participation resembles a more active contribution of stakeholders in decision-making. For a more detailed overview see Hanssen & Gremmen (2013).

4 All authors of this paper are or were members of the subcommittee on Ethics and Societal Aspects (ScEMA) of the Netherlands Commission on Genetic Modification (COGEM). In this role, we often took (and take) part in or observe activities related to stakeholder consultation or participation. We often witnessed frustrations among stakeholders and even a deadlock of debate. Stakeholders felt ignored or not taken seriously as they expected to participate in developing a new policy. In fact, it was not about challenging viewpoints and working together towards a new policy.

5 Zinc finger nuclease (ZFN) technology (ZFN-1, ZFN-2 and ZFN-3), Oligonucleotide directed mutagenesis, Cisgenesis and intragenesis, RNA-dependent DNA methylation, Grafting (on GM rootstock), Reverse breeding, Agro-infiltration, Synthetic genomics (synthetic biology).

6 The questionnaire: https://ec.europa.eu/food/plant/gmo/modern_biotech/stakeholder-consultation_en

7 The term ‘comitology’ refers to the set of procedures through which the European Commission exercises the implementing powers conferred on it by the EU legislator, with the assistance of committees of representatives from EU countries. Such comitology committees are chaired by a Commission official and give an opinion on implementing acts proposed by the Commission.

8 See for example: GM Watch, see: https://www.gmwatch.org/en/news/latest-news/19719-eu-commission-breaking-own-rules-to-give-green-light-for-new-gmos; Friends of the Earth, see: https://friendsoftheearth.eu/press-release/eu-commission-backs-removing-safety-checks-for-new-gmos/ IFOAM Organics Europe, see: https://my.organicseurope.bio/civicrm/mailing/view?reset=1&id=3987

9 All authors worked together in a working group initiated by COGEM (see also Footnote 4) to explore the conditions for effective stakeholder involvement. This exploration resulted in distinction between two stages of stakeholder involvement in the field of genomic modification of animals: one stage on structuring the problem at stake by articulation and confrontation of diverse considerations; and a second stage in which possible solutions are identified and evaluated. In the context of GM animals, we identified a broad range of subjects relating to a diversity of stakeholders and goals of involvement. See *CRISPR & Animals: Implications of Genome Editing for Policy and Society*, COGEM 2018.

10 In our line of argument participants refer to stakeholders in a wide sense, including publics with immaterial interests.

11 The questionnaire: https://ec.europa.eu/food/plant/gmo/modern_biotech/stakeholder-consultation_en
